# MSS-MambaNet: A Mamba Framework for Building Extraction from Multi-Phase Disaster Imagery

**DOI:** 10.3390/s26123868

**Published:** 2026-06-17

**Authors:** Xin Liang, Huijiao Qiao, Yanda Chen, Jin Zhang

**Affiliations:** 1 Taiyuan University of Technology, Taiyuan 030024, China; 2023005811@link.tyut.edu.cn (X.L.); 2024005338@link.tyut.edu.cn (Y.C.); zjgps@163.com (J.Z.); 2 China Coal Technology & Engineering Group, Taiyuan Research Institute, Taiyuan 030006, China

**Keywords:** building extraction, multi-phase data, natural disaster, Mamba, multi-scale

## Abstract

Building extraction from disaster scenes is critical for emergency response and post-disaster assessment. Unlike conventional static remote sensing imagery, multi-phase disaster imagery contains scenes spanning early, middle, and late disaster stages, where building morphology, class distribution, and boundary characteristics exhibit significant cross-phase heterogeneity. Such phase-dependent variations substantially increase the difficulty of stable semantic segmentation, particularly under complex damage conditions. To address these challenges, we propose MSS-MambaNet for building extraction from multi-phase disaster imagery. A multi-scale architecture is designed to overcome the limitations of single-scale scanning in Mamba, enabling more effective perception of diverse building morphologies. To enhance feature discrimination, a Dual-Domain Cross-Gated Fusion (DDCGF) module is introduced through complementary interactions between spatial and frequency-domain representations. In addition, a Pixel-Aware Dynamic Weighting (PADW) strategy is developed to adaptively emphasize imbalanced foreground pixels and ambiguous boundary regions, thereby improving segmentation consistency under complex disaster conditions. Extensive experiments demonstrate that MSS-MambaNet consistently outperforms state-of-the-art methods, achieving an average mIoU of 92.78% and mF1 of 96.25% with only 12.37 M parameters. These results indicate that the proposed method effectively handles the heterogeneity of multi-phase data, providing a stable and efficient solution for building extraction from multi-phase disaster imagery.

## 1. Introduction

The frequency and intensity of natural disasters have risen markedly worldwide, resulting in severe economic losses and widespread human displacement. Accurate and timely building extraction from remote sensing data is essential for disaster monitoring, emergency response, and damage assessment [1,2,3].

Traditional methods typically extract buildings by designing handcrafted features—such as spectral, spatial, and texture cues—and feeding them into simple classifiers such as support vector machines and random forests [4,5,6]. In recent years, deep learning has emerged as the dominant paradigm for building extraction from remote sensing imagery.

CNN-based methods have made substantial progress in capturing multi-scale features and refining building boundaries. However, the local receptive field inherent to convolution limits their capacity to model long-range dependencies, which are essential for segmenting large-scale or occluded buildings [7,8,9].

To overcome this limitation, researchers have introduced Transformer architectures [10], which excel at global context modeling through self-attention mechanisms. Transformer-based methods [11,12,13] have demonstrated superior performance in complex scenarios but often incur quadratic computational complexity with respect to sequence length. These advances have been extended to post-disaster scenarios, where accurate building extraction is critical for damage assessment and rescue planning.

Recognizing the complementary strengths of CNNs and Transformers, recent studies have explored hybrid architectures that integrate both paradigms. For instance, BCTNet [14] employs a dual-branch cross-fusion Transformer to improve building boundary delineation by integrating global context with local details; EasyNet [15] utilizes a lightweight hybrid CNN–Transformer framework with task-specific feature fusion strategies for efficient building extraction, and MC-TRANSU [16] adopts a serialized encoder combining CNN and Transformer modules with multi-scale constrained attention to leverage both local and global features.

Beyond CNN- and Transformer-based architectures, state-space models (SSMs), particularly Mamba [17], have recently emerged as a promising alternative for visual data processing. Mamba introduces a selective state-space mechanism that dynamically modulates information flow according to the input, enabling effective capture of long-sequence dependencies while suppressing redundant information and thereby achieving a favorable balance between modeling capacity and computational efficiency [18]. Studies have demonstrated its strong adaptability across video understanding, long-sequence modeling, remote sensing analysis, and high-resolution vision tasks [19]. However, Mamba was originally designed for 1D sequences. Extending its selective scanning mechanism to 2D visual data typically requires flattening the spatial structure into a 1D sequence, which disrupts the local spatial coherence and hierarchical relationships inherent in images and impairs the capture of multi-scale spatial structures. To mitigate this issue, Vision Mamba [20] and VMamba [21] introduced bidirectional and four-way cross-scanning strategies, respectively, extending 1D state-space modeling to the 2D spatial domain. Within the specific task of building an extraction from remote sensing imagery, researchers have further explored the applicability of Mamba. Zhao et al. [22] proposed MDA-RSM, which introduces a multi-directional adaptive scanning mechanism to capture irregular building geometries. Zhou et al. [23] proposed Mamba-in-Mamba with a Centralized Mamba-Cross-Scan mechanism, which transforms images into diverse half-directional scanning paths to better preserve spatial continuity in remote sensing images.

Although these methods have improved Mamba’s adaptability to visual data, they mainly add scanning directions within a fixed scanning paradigm. Linear or raster scanning disrupts local spatial structures, while bidirectional or multi-directional scanning, despite enhancing spatial representation, significantly increases computational overhead without guaranteeing consistent performance gains [24,25]. In other words, the multi-scale modeling capability of these algorithms is constrained by a predefined receptive field, making it difficult to capture both global context and local details simultaneously. Notably, this limitation does not contradict Mamba’s established strengths. Mamba’s ability to efficiently model long-range dependencies along a sequence remains intact. The issue arises only when adapting the 1D scanning mechanism to 2D visual data, where preserving spatial topology and hierarchical feature relationships requires fundamentally different processing [26]. This limitation is especially acute in multi-phase disaster scenarios, where buildings undergo continuous transformation from intact structures to complete fragmentation. Effectively modeling such scenes requires simultaneous capture of large-scale structural features and fine-grained debris characteristics, placing substantially greater demands on multi-scale feature extraction.

Morphological diversity is only one aspect of the heterogeneous data distribution in multi-phase satellite imagery of disaster scenes. Notably, images from the early, middle, and late stages of a disaster exhibit significant disparities in class proportions and boundary clarity. Owing to the macroscopic scale of remote sensing imagery, land cover classes are inherently imbalanced in most scenes. As a result, class imbalance is a pervasive issue that considerably constrains the training accuracy and generalization capability of classifiers [27]. The most straightforward strategy for addressing class imbalance is data resampling, which balances the class distribution by over-sampling the minority class or under-sampling the majority class. However, over-sampling often leads to overfitting on minority class samples, whereas under-sampling risks discarding majority class samples that carry valuable information [28]. A more effective alternative is to incorporate reweighting or modulating factors directly into the loss function. This strategy sharpens the model’s focus on minority classes and hard examples during training, without the information redundancy or overfitting risks inherent to resampling. For instance, Focal Loss [29] modulates sample weights through a dynamic focusing factor, and Class-Balanced Loss [30] reweights classes based on effective sample numbers. In the field of remote sensing-based building segmentation and damage assessment, the xBD dataset is widely recognized as a benchmark [31]. This dataset suffers from severe class imbalance, where samples labeled as severely damaged or completely destroyed are far outnumbered by those labeled as undamaged. To address this issue, Gupta et al. [32] proposed a cost-sensitive reweighting strategy that assigns higher weights to minority classes, effectively improving the model’s ability to identify critically damaged buildings. To mitigate the foreground–background imbalance caused by building pixels being substantially outnumbered by background pixels, Ge et al. [33] designed a unified Focal Loss. Their work further validated the effectiveness of loss function-based solutions.

In remote sensing building extraction, boundary blurring is a common issue due to factors such as image resolution and object occlusion. A proven remedy is to design boundary-aware loss functions that penalize discrepancies between predicted and ground-truth boundaries. The boundary loss proposed by Bokhovkin et al. [34] improved building boundary accuracy on the ISPRS Potsdam and INRIA AIL datasets. Hosseinpour et al. [35] further designed a derivative boundary loss tailored to high-resolution remote sensing imagery, optimizing building boundary extraction through distance transform images. For building contour regularization, Hu et al. [36] proposed a boundary shape-preserving model that smooths building boundary noise via a boundary loss.

To jointly address the coupled challenges of class imbalance and boundary blurring, recent studies have explored composite loss functions. For instance, Mortazavi et al. [37] combined BCE, Tversky, and Edge-Weighted Hinge losses to handle both challenges simultaneously. Sheikh et al. [38] proposed BCEDL, where Cross-Entropy and Dice Loss account for global and local information, respectively, reducing the impact of class imbalance while improving building boundary extraction. While these methods perform well on conventional remote sensing imagery, they share a tacit assumption: that class proportions and boundary clarity remain relatively stable across the dataset. The data used in this study breaks this assumption. The dataset contains images from three distinct disaster phases, namely early, middle, and late, which exhibit divergent building morphologies, class proportions, and degrees of boundary blur.

When applied to such strongly heterogeneous data, the inherent static assumptions of existing methods bias the model toward the dominant distribution patterns present in the training data. This bias manifests in two complementary ways. Static reweighting strategies rely on fixed weights computed from global class statistics. However, class proportions vary considerably across the three disaster phases. A single set of fixed weights cannot simultaneously accommodate the distinct imbalance characteristics of each phase, from the relatively balanced early stage to the severely imbalanced late stage. Boundary-aware losses, in turn, assume that boundary clarity remains consistent throughout the dataset. As a consequence, these models become adept at handling the sharp boundaries characteristic of the early stage, while their performance on the highly blurred boundaries of the late stage remains limited. Even when late-stage data is included during training, the model continues to exhibit bias toward the dominant distribution characteristics of the early-stage data. Therefore, existing methods demonstrate clear limitations when applied to strongly heterogeneous data such as multi-phase disaster imagery. This underscores the need for a loss function capable of adaptively adjusting pixel-wise weights and accommodating multiple coexisting distribution characteristics. Recent advances in cross-domain remote sensing analysis have further underscored the importance of modeling distribution shifts beyond static class assumptions. SpectralX [39] and BiDA [40] address parameter-efficient domain generalization and cross-domain hyperspectral classification, respectively, demonstrating that heterogeneous imaging conditions require adaptive rather than static modeling strategies. From a temporal perspective, UniTS [41] proposes a unified spatio-temporal generative framework for remote sensing time series, highlighting the value of jointly modeling multi-phase observations. These studies motivate our PADW strategy, which explicitly accounts for phase-specific distribution characteristics through pixel-level dynamic weighting.

In summary, we propose a Multi-scale Mamba Network (MSS-MambaNet) for building extraction from multi-phase disaster imagery, addressing the limitations of Mamba-based models and cross-phase distribution heterogeneity. To alleviate the structural limitations of Mamba, this paper designs the MSS-Mamba backbone with a global–local dual-path architecture. The global path introduces an attention mechanism into Mamba’s SS2D scanning to enhance targeted global context modeling. The local path employs parallel multi-scale convolutions to extract multi-scale contextual features and introduces a scale-adaptive weighting mechanism that dynamically adjusts the contribution of features from different convolutional kernels according to the degree of building damage, thereby adaptively allocating appropriate receptive fields to buildings of varying scales. This architecture preserves linear computational complexity while capturing both long-range dependencies and fine-grained spatial features. To further enhance feature interaction between the encoder and decoder, this paper proposes the Dual-Domain Cross-Gated Fusion (DDCGF) module, achieving complementary fusion of spatial and frequency-domain features. The module extracts spatial features via Spatial Context Refinement and frequency-domain features via Spectral Detail Enhancement, then fuses them through a cross-gated mechanism that balances multi-scale and cross-domain contributions to the final output. In addition, we introduce a pixel-aware, dynamically weighted loss function to address the dynamic class imbalance and edge degradation caused by cross-phase variations in class proportions and boundary clarity in multi-phase disaster imagery. This loss function adaptively adjusts sample weights based on disaster phase priors and local pixel statistics. It enables the model to continuously focus on hard example regions characterized by blurred boundaries and fragmented structures within each phase during training, thereby enhancing the overall learning effectiveness across the three heterogeneous data distributions of the early, middle, and late phases. By simultaneously addressing the architectural limitations of Mamba, insufficient feature fusion, and statistical data heterogeneity, the proposed method achieves consistent building extraction across different disaster phases without incurring additional computational overhead.

The remainder of this paper is organized as follows. Section 2 details the proposed MSS-MambaNet. Section 3 presents experimental results and comparisons with state-of-the-art methods. Section 4 discusses the findings and outlines future directions.

## 2. Materials and Methods

### 2.1. Overview of the Proposed Framework

**Figure 1:**
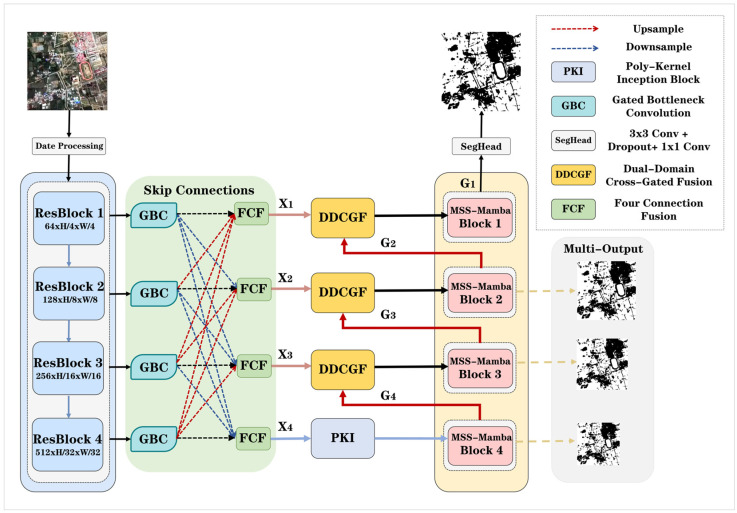
Framework of the proposed MSS-MambaNet.

As illustrated in Figure 1, MSS-MambaNet adopts a U-shaped encoder–decoder architecture tailored to the progressive structural degradation characteristic of disaster scenes, in which building morphology varies drastically across stages.

A four-stage encoder first extracts hierarchical features at multiple spatial resolutions. Gated Bottleneck Convolution units are incorporated for efficient representation recalibration, ensuring feature consistency across scales. The resulting multi-scale features are aligned and fused via a four-connection strategy that facilitates cross-scale interaction, yielding a comprehensive spatial–semantic representation across resolutions.

The decoder adopts a progressive refinement strategy from deep to shallow levels. At the deepest stage, a Poly Kernel Inception module [42] provides initial semantic enhancement. The refined representation then passes through the MSS-Mamba blocks, which serve as the core modeling units, capturing long-range dependencies with linear complexity while preserving structural details. At the remaining stages, DDCGF modules iteratively refine features prior to global modeling, jointly exploiting spatial and frequency-domain information to better represent ambiguous and fragmented building regions. This refinement–modeling process progressively strengthens feature discriminability from coarse to fine scales. Finally, a Pixel-Aware Dynamic Weighting Loss is employed to jointly mitigate severe, phase-dependent class imbalance and boundary ambiguity, especially in degraded mid- and late-phase scenes. In this way, MSS-Mamba, DDCGF, and PADW correspond respectively to morphological variation, feature fusion inconsistency, and phase-dependent optimization difficulty.

### 2.2. Multi-Scale Mamba Block

To handle extreme scale variations and complex contextual interference across disaster phases, we propose the Multi-Scale Mamba (MSS-Mamba) Block. It integrates long-range dependency modeling with adaptive multi-scale spatial feature extraction within a unified framework, enabling consistent segmentation from intact structures to severely degraded remnants.

**Figure 2:**
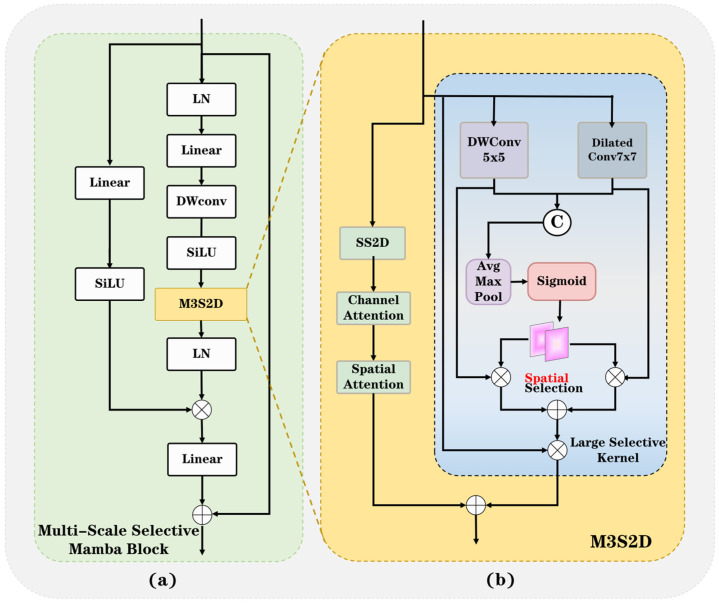
Architecture of the proposed MSS-Mamba backbone. (**a**) Structure of the MSS-Mamba block, which serves as the fundamental building unit of the backbone. (**b**) Internal design of the M3S2D module, illustrating the dual-pathway architecture composed of the enhanced SS2D Global Pathway and the LSK-based Local Pathway.

As illustrated in Figure 2, given an input feature map $Y\in R^{H\times W\times C}$ the MSS-Mamba block first performs channel projection and lightweight spatial encoding. The processed features are then fed into a core sub-module termed Multi-Scale Selective Scan 2D (M3S2D) for jointly modeling global and local features. In parallel, a lightweight gating branch generates a modulation signal from the original input features, which adaptively gates the M3S2D output through element-wise multiplication. Finally, a linear projection restores the channel dimension, and a residual connection with Y produces the final output G.

Formally, the overall computation of the MSS-Mamba block can be expressed as:

(1) $$X_{1}=LN(M3S2D(SiLU(DWConv(Linear(Y)))))$$

(2) $$X_{2}=SiLU(Linear(Y))$$

(3) $$G=Linear(X_{1}⊙X_{2})+Y$$

where LN denotes Layer Normalization and ⊙ denotes element-wise multiplication.

The M3S2D module adopts a dual-pathway architecture (Figure 2b) that tightly couples global dependency modeling with adaptive multi-scale spatial representation. The two pathways operate in a complementary and interactive manner, rather than as independently stacked modules. The Enhanced 2D-Selective-Scan (SS2D) Global Pathway with Cascaded Channel–Spatial Attention captures long-range dependencies, while the Large Selective Kernel (LSK) [43] Local Pathway with Adaptive Multi-Scale Fusion extracts fine-grained spatial details. Their outputs are fused through element-wise addition, producing a representation that integrates global semantics with local structural detail.

#### 2.2.1. Enhanced SS2D Global Pathway with Cascaded Channel–Spatial Attention

The global pathway within the M3S2D module is built upon the SS2D mechanism. SS2D efficiently models long-range spatial dependencies with linear complexity by performing sequential scans in four directions—top-down, bottom-up, left-right, and right-left—a capability that is crucial for understanding the global layout of a disaster scene. Through its selective mechanism, SS2D adaptively adjusts its parameters based on the input, enabling dynamic, input-dependent computation. However, the output of standard SS2D is a global, isotropic blend of information. While effective for holistic context modeling, this representation may fail to emphasize the specific channels and spatial locations most discriminative for identifying building structures in complex disaster scenes.

To address this limitation, we introduce a Cascaded Channel–Spatial Attention mechanism to refine the SS2D representations. This mechanism adopts a two-stage refinement strategy. First, a channel attention (CA) module assigns importance weights to each feature channel, highlighting informative responses while suppressing noise. Then, a Spatial Attention (SA) module emphasizes discriminative spatial locations to further refine structural representations.

Given an input feature $X_{global}\in R^{C\times H\times W}$ from the SS2D pathway, the CA module first aggregates spatial information to generate a channel-wise weighting feature:

(4) $$X_{c}=\underset{CA}{\underbrace{\sigma (W_{1}(W_{0}({GAP}_{c}(X_{global}),{GMP}_{c}(X_{global}))))}}⊙X_{global}$$

where ${GAP}_{c}(\cdot )$ and ${GMP}_{c}\left(\cdot \right)$ denote the results of global average pooling and global max pooling, respectively. $W_{0}$ and $W_{1}$ represent the weights of the fully connected layers, while σ refers to the sigmoid function.

Subsequently, the SA module operates on $X_{c}$. We innovatively apply channel-wise global average pooling and max pooling to generate two spatial descriptors, which are concatenated and processed by a convolutional layer to produce a spatial weight map. The final output of the global pathway is:

(5) $$X_{SS2D}=\underset{SA}{\underbrace{\sigma ({Conv}_{7\times 7}[F_{avg}(X_{c});F_{max}(X_{c})])}}⊙X_{c}$$

where the notation ${Conv}_{7\times 7}(\cdot )$ indicates a standard convolution operation with a kernel size of 7×7. The expression $\left[F_{avg}(\cdot );F_{max}(\cdot )\right]$ signifies the concatenation of the results from channel-wise average pooling and channel-wise max pooling along the channel dimension.

#### 2.2.2. LSK-Based Local Pathway with Adaptive Multi-Scale Fusion

To complement the global pathway in capturing multi-scale local details, the M3S2D module integrates an LSK module as a parallel local pathway. The core idea of LSK is to achieve dynamic receptive field selection via parallel convolutions and adaptive weighting. In this work, we adapt it as an efficient multi-scale local feature extractor. Specifically, the input feature Y′ is processed in parallel by two depth-wise convolutional branches: a standard 5×5 depth-wise convolution for fine-grained details, and a 7×7 dilated depth-wise convolution with a dilation rate of 3 for broader contextual information.

(6) $$F_{1}={Conv}_{1\times 1}({DWConv}_{5\times 5}(Y\prime ))$$

(7) $$F_{2}={Conv}_{1\times 1}({DWConv}_{7\times 7}^{d=3}(Y\prime ))$$

where DWConv denotes depth-wise convolution, ${DWConv}_{7\times 7}^{d=3}$ represents dilated convolution with kernel size 7×7 and dilation rate 3.

To achieve adaptive fusion, a spatial selection gate generates soft attention maps $w_{1}$ and $w_{2}$ from the concatenated multi-scale features. These maps perform pixel-wise weighting of the corresponding feature maps $F_{1}$ and $F_{2}$ before summation, enabling content-aware integration of multi-scale details. This formula computes the final output by using the input-adapted, multi-scale features to perform a gated refinement of the input feature Y′.

(8) $$F_{concat}=Concat(F_{1},F_{2})$$

(9) $$w_{1},w_{2}=\sigma ({Conv}_{7\times 7}[F_{avg}(F_{concat});F_{max}(F_{concat})])$$

(10) $$X_{LSK}=Y\prime \cdot (w_{1}⊙F_{1}+w_{2}⊙F_{2})$$

where $w_{1},w_{2}\in R^{H\times W}$ are the adaptive attention weights.

The outputs from the SS2D and LSK branches are fused through element-wise addition, enabling the model to combine global semantic context and multi-scale local features.

(11) $$X_{M3S2D}=X_{LSK}+X_{SS2D}$$

Through this fusion strategy, the M3S2D module integrates the global contextual information captured by enhanced SS2D with the multi-scale local features extracted by LSK. The two branches process the same input through separate transformation paths: enhanced SS2D focuses on long-range semantic dependency modeling, while LSK emphasizes local multi-scale structural details. Their branch-specific features are fused only after independent transformation, which helps reduce direct interference between global and local representations. This complementary representation enables the model to capture both overall scene structure and fine-grained building details, thereby improving segmentation performance for damaged buildings with diverse scales and irregular shapes in complex disaster environments.

### 2.3. Dual-Domain Cross-Gated Fusion Module

**Figure 3:**
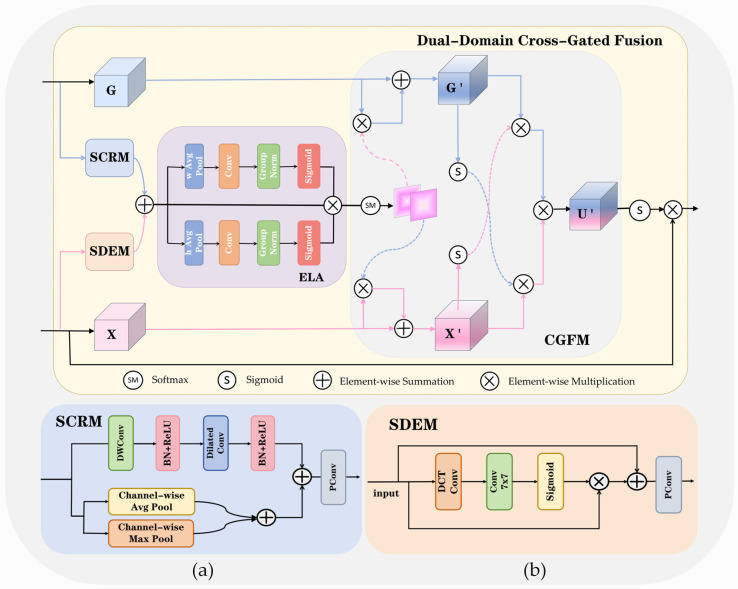
The overall architecture of the Dual-Domain Cross-Gated Fusion Module. (**a**) Spatial Context Refinement Module. (**b**) Spectral Detail Enhancement Module.

To enhance encoder–decoder feature interaction, we propose the DDCGF module that leverages complementary spatial and frequency-domain optimization. As illustrated in Figure 3, DDCGF consists of three key components: (1) a Spatial Context Refinement Module (SCRM), (2) a Spectral Detail Enhancement Module (SDEM), and (3) a Cross-Gated Fusion Mechanism (CGFM) for adaptive fusion.

Given encoder features X and decoder features G, DDCGF first processes them in parallel across two domains. The spatial branch focuses on contextual semantic refinement to strengthen structural continuity and boundary consistency, while the frequency branch enhances high-frequency structural details and suppresses noise interference. The outputs from both branches are then fused and enhanced through the Enhanced Local Attention (ELA) mechanism, which adaptively weights the fused features to highlight discriminative patterns. The enhanced features are projected into a shared embedding space and passed through a cross-gated mechanism that adaptively regulates information flow between the encoder and decoder.

Through this dual-domain design, DDCGF explicitly separates spatial and spectral modeling prior to gated interaction, reducing feature interference and promoting complementary enhancement across domains.

#### 2.3.1. Spatial Context Refinement Module (SCRM)

The SCRM enhances spatial structural coherence by jointly modeling local continuity and global context through a dual-branch architecture (Figure 3a). Given an input feature $G\in R^{C\times H\times W}$, the local branch employs two cascaded depth-wise 3×3 convolutions (${DWConv}_{3\times 3}$) with progressively enlarged receptive fields, where the second convolution adopts a dilation rate d=2 to enlarge the effective receptive field. Each convolution is followed by batch normalization and ReLU activation, capturing local structural patterns at multiple scales.

The global context branch applies channel-wise average pooling (${GAP}_{c}$) and channel-wise max pooling (${GMP}_{c}$) to produce spatial attention descriptors that capture global context. These operations aggregate information across the channel dimension to yield a global descriptor of shape $R^{1\times H\times W}$, which is then broadcast to match the dimensions of the local features. The local and global representations are fused through element-wise addition, followed by a 1×1 convolution with batch normalization that reduces the channel dimension to C/2, achieving efficient feature compression while preserving spatial coherence. The complete process can be formulated as:

(12) $$F_{SCRM}=BN({Conv}_{1\times 1}(\underset{local}{\underbrace{{DWConv}_{3\times 3}^{d=2}({DWConv}_{3\times 3}(G))}}+(\underset{global}{\underbrace{{GAP}_{c}(G)+{GMP}_{c}(G)}}))$$

where $F_{SCRM}\in R^{C/2\times H\times W}$ denotes the output feature.

By decoupling local structural modeling from global contextual aggregation, SCRM effectively integrates multi-scale spatial information while maintaining computational efficiency.

#### 2.3.2. Spectral Detail Enhancement Module (SDEM)

The SDEM enhances high-frequency structural details and suppresses noise interference by leveraging frequency-domain analysis (Figure 3b). Given an input feature $X\in R^{B\times C\times H\times W}$, SDEM first performs frequency decomposition using a set of learnable Discrete Cosine Transform (DCT) filters. Let $W_{DCT}\in R^{N\times C\times K\times K}$ denote the DCT filter bank, where N=49 represents the number of frequency components, and K is the filter kernel size. Through the DCT-based decomposition, the input feature is transformed into frequency-domain representations with N channels, each capturing structural information at a specific frequency band. This multi-scale spectral representation enables the module to distinguish between informative high-frequency structures and noise.

To selectively enhance the valuable frequency components while suppressing irrelevant noise, the module generates a frequency attention map from these spectral responses via a 7×7 convolution with padding p=3 followed by sigmoid activation. This attention map assigns adaptive spatial weights to highlight discriminative frequency patterns. The attention weights are then broadcast to match the input channel dimension and applied to the original feature X through element-wise multiplication, combined with a residual connection to preserve base feature information. Finally, a 1×1 convolution followed by batch normalization reduces the channel dimension to $R^{C/2\times H\times W}$ achieving efficient feature compression while maintaining representational capacity. The process is formulated as:

(13) $$F_{SDEM}=BN({Conv}_{1\times 1}(X⊙Expand\left(\sigma \left({Conv}_{7\times 7}^{p=3}\left(Conv2D(X,W_{DCT})\right)\right)\right)+X))$$

where $F_{SDEM}\in R^{C/2\times H\times W}$ denotes the final output feature.

By decoupling frequency decomposition from adaptive residual modulation, SDEM learns to strengthen informative structural details while suppressing noise-prone frequency responses, thereby reducing false edges caused by debris or artifacts.

#### 2.3.3. Cross-Gated Fusion Mechanism (CGFM)

To achieve deep cross-domain interaction between encoder and decoder features, we propose a CGFM. Instead of conventional cross-attention, CGFM employs a bidirectional cross-gated modulation mechanism that enables mutual feature gating between encoder and decoder streams, forming the core of our interaction strategy.

(1) Spatial refinement via ELA

After fusing the spatial and frequency branch outputs via element-wise addition followed by batch normalization, the Efficient Localization Attention (ELA) [44] module is applied to refine spatial localization. By modeling global contextual dependencies along horizontal and vertical directions, ELA produces spatial attention weights that recalibrate the fused feature map. The resulting representation is denoted as $U\in R^{C/2\times H\times W}$, which serves as the refined context for generating the subsequent modulation maps.

(2) Initial feature modulation

To adaptively balance encoder and decoder contributions, the refined feature ***U*** is projected through a 1×1 convolution followed by channel-wise softmax normalization, as result of yielding two spatial attention maps $W_{1},W_{2}\in R^{1\times H\times W}$ with $\sum_{i=1}^{2}W_{i}=1$:

(14) $$W_{i}=Softmax\left({Conv}_{1\times 1}\left(U\right)\right),\;\;\forall i\in [1,2]$$

These weights recalibrate the encoder feature $X\in R^{C\times H\times W}$ and decoder feature $G\in R^{C\times H\times W}$ through residual modulation:

(15) $$X\prime =W_{1}⊙X+X,\;\;\;\;G\prime =W_{2}⊙G+G$$

(3) Bidirectional Cross-Gated Modulation

While the above attention mechanism provides spatially adaptive weighting, it does not explicitly model inter-branch dependency. To address this limitation, we propose a bidirectional cross-gated modulation strategy that enables mutual guidance between encoder and decoder streams.

Specifically, each branch is dynamically modulated by the other through sigmoid-gated interactions:

(16) $$X\prime \prime =X\prime ⊙\sigma \left(G\prime \right),\;\;\;\;G\prime \prime =G\prime ⊙\sigma (X\prime )$$

where X″ and $G\prime \prime \in R^{C\times H\times W}$ denote the cross-modulated features.

This symmetric gating establishes a bidirectional information flow in which each branch acts as a context-aware filter for the other, enabling fine-grained feature selection and deeper cross-domain interaction beyond conventional fusion strategies such as addition or concatenation.

The modulated features are integrated via element-wise interaction:

(17) U′=X″⊙G″

where $U\prime \in R^{C\times H\times W}$ represents the comprehensive cross-domain fusion.

Finally, the bidirectionally modulated feature U′ is first projected through a 1×1 convolution followed by batch normalization and sigmoid activation to generate a spatial-channel attention map. This attention map recalibrates the original encoder feature X via element-wise multiplication. The recalibrated feature is then passed through a second 1×1 convolution with batch normalization to produce the final output:

(18) $$Y=BN({Conv}_{1\times 1}(\sigma (BN({Conv}_{1\times 1}(U\prime )))⊙X))$$

where $Y\in R^{C\times H\times W}$ denotes the final fused representation.

### 2.4. Pixel-Aware Dynamic Weighting Loss (PADW)

Post-disaster building segmentation faces two critical challenges: severe foreground–background imbalance and progressive blurring of building boundaries, both of which vary significantly across disaster phases. Conventional loss functions with fixed class weights and static regularization terms cannot adapt to such dynamic distributions. This limitation leads to model bias toward majority classes and coarse boundary predictions in severely imbalanced and blurred late-phase scenes. To address this, we propose a PADW loss, which adaptively adjusts sample weights based on both pixel-level class imbalance ratios and disaster-phase context. By dynamically emphasizing difficult samples from minority classes and ambiguous boundary regions, PADW enables more balanced learning across different disaster stages. The proposed PADW loss is constructed in two steps: the dynamic weight computation strategy and the formulation of the final loss function integrating the adaptive weights.

#### 2.4.1. Dynamic Weight Computation

For each training sample, the proposed PADW loss computes a pixel-aware dynamic weight that accounts for both pixel-level class imbalance and disaster phase context. The dynamic weight $w_{dy}^{\varphi_{b}}$ is defined as:

(19) $$w_{dy}^{\varphi_{b}}=w_{base}^{\varphi_{b}}\times w_{phase}^{\varphi_{b}}$$

where $w_{base}^{\varphi_{b}}$ reflects the imbalance between background and building pixels, and $w_{phase}^{\varphi_{b}}$ is a phase-specific factor that captures the varying difficulty across disaster stages.

To quantify pixel-level imbalance, we compute the class imbalance ratio for each sample, based on which the base dynamic weight is computed using a square-root strategy.

(20) $$w_{base}^{\varphi_{b}}=\sqrt{\frac{N_{bg}^{\varphi_{b}}}{N_{bd}^{\varphi_{b}}}}$$

where $\varphi_{b}\in [0,1,2]$ corresponds to the early, middle, and late phases, and $N_{bg}^{\varphi_{b}}$ and $N_{bd}^{\varphi_{b}}$ denote the number of background pixels and building pixels, respectively.

(21) $$N_{bg}^{\varphi_{b}}=\sum_{i,j}I[Y_{i,j}^{\varphi_{b}}=l_{bg}],\;\;\;\;N_{bd}^{\varphi_{b}}=\sum_{i,j}I[Y_{i,j}^{\varphi_{b}}=l_{bd}]$$

where $l_{bd}$ and $l_{bg}$ denote the label indices for building and background classes, respectively. $Y_{i,j}^{\varphi_{b}}$ represents the ground-truth label at pixel location (i,j), and I[⋅] is the indicator function that returns 1 if the condition is true and 0 otherwise.

In addition, disaster phases introduce different levels of segmentation difficulty. To account for this variation, we introduce phase-specific weighting factors:

(22) $$w_{phase}^{\varphi_{b=0}}=0.8,\;\;w_{phase}^{\varphi_{b=1}}=1.5,\;\;w_{phase}^{\varphi_{b=2}}=2.5$$

These weighting factors were determined through repeated preliminary experiments with different phase-weight combinations. The selected configuration provided a stable balance between emphasizing difficult degraded-phase samples and avoiding excessive weighting of noisy fragmented regions in the late phase.

Finally, the dynamic weight ($w_{dy}^{\varphi_{b}}$) integrates information on both pixel distribution imbalance and disaster phase context, thereby assigning higher weights to samples from phases that are severely imbalanced and exhibit highly blurred boundaries.

#### 2.4.2. Loss Function Formulation

The proposed PADW loss combines a phase-aware Focal Loss ${\;(L}_{PAF})$ and a phase-aware Dice Loss ${(L}_{PAD})$ for each segmentation prediction map:

(23) $$L_{PADW}=L_{PAF}+L_{PAD}$$

The $L_{PAF}$ applies dynamic weights exclusively to building pixels within each batch:

(24) $$L_{PAF}=\frac{1}{\left|\Omega \right|}\sum_{b=1}^{B}\sum_{i,j\in \Omega }w_{dy}^{\varphi_{b}}\cdot L_{focal}^{\varphi_{b}}\cdot I[Y_{i,j}^{\varphi_{b}}=l_{bd}]$$

(25) $$L_{focal}^{\varphi_{b}}=-\alpha (1-p_{t}^{\varphi_{b}}{)}^{\gamma }\log(p_{t}^{\varphi_{b}})$$

where |Ω| is the total number of valid pixels in the batch, B is the batch size, α balances class priors, and γ controls the focusing strength, $p_{t}^{\varphi_{b}}$ denotes the predicted probability for the true class.

To further optimize region-level overlap, the $L_{PAD}$ introduces dynamic weights to the building class at the category level:

(26) $$L_{PAD}=\frac{1}{B}\sum_{b=1}^{B}w_{dy}^{\varphi_{b}}\cdot L_{dice}^{\varphi_{b}}$$

(27) $$L_{dice}^{\varphi_{b}}=1-\frac{2\sum_{i,j}P_{1,i,j}^{\varphi_{b}}Y_{i,j}^{\varphi_{b}}+1}{\sum_{i,j}P_{1,i,j}^{\varphi_{b}}+\sum_{i,j}Y_{i,j}^{\varphi_{b}}+1}$$

where $P_{1,i,j}^{\varphi_{b}}$ represents the predicted probability for the building class after softmax. The numerator and denominator are smoothed by adding 1 for numerical stability.

#### 2.4.3. Total Loss with Auxiliary Supervision

The total loss during the training phase is defined as:

(28) $$L_{total}=L_{PADW}(P_{main},Y,\varphi_{b})+\lambda_{aux}\cdot \frac{1}{K}\sum_{k=1}^{K}L_{PADW}(P_{aux}^{k},Y,\varphi_{b})$$

where $P_{main}$ denotes the main segmentation prediction, $P_{aux}^{k}$ denotes the auxiliary predictions and $\frac{1}{K}$ is the normalization factor, $\lambda_{aux}$ is the auxiliary loss coefficient, where *λ* is the auxiliary loss coefficient, set to 0.4. This moderate coefficient provides additional gradient guidance for intermediate decoder features while preventing the auxiliary objective from dominating the main segmentation loss.

The auxiliary supervision encourages intermediate decoder features to learn meaningful semantic representations, which facilitates optimization and improves training stability. During inference, only the main prediction $P_{main}$ is used for segmentation. Overall, the PADW loss integrates phase-aware dynamic weighting with multi-level supervision, enabling stable optimization under severe and phase-varying class imbalance and boundary degradation.

## 3. Experiment and Result Analysis

### 3.1. Datasets

The dataset used in this study consists of satellite video acquired by the DigitalGlobe WorldView satellite during the tsunami event in Petobo, Indonesia, which was triggered by a magnitude 7.5 earthquake on 28 September 2018. The video provides continuous observations of the affected area throughout the disaster process. The original video was first converted into a sequence of individual frames. After preprocessing to remove invalid segments, such as introductory clips and artificially inserted buffer frames, a total of 301 effective key frames were retained as the basis for subsequent building extraction. Ground-truth labels were generated through SVM-assisted classification and manual visual refinement, with annotation reliability confirmed by an overall accuracy above 97% and a Kappa coefficient above 95.50%.

**Figure 4:**
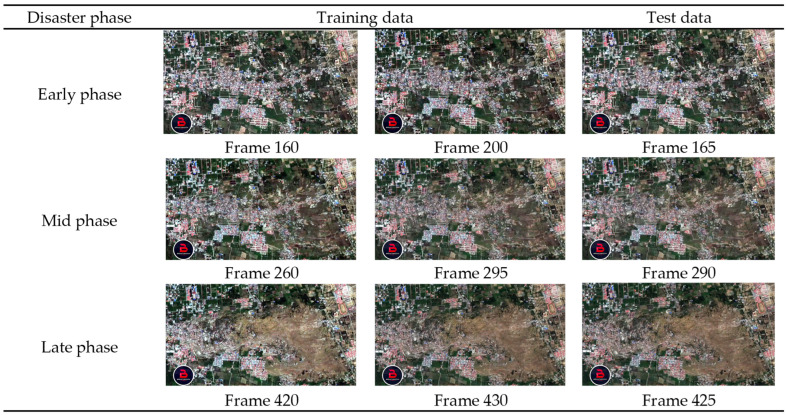
Experimental data.

The tsunami process can be divided into three temporal phases according to the degree of structural impact: early, mid, and late phases. The early phase corresponds to the initial stage of the disaster, during which structural changes are limited and only slight displacements are observed. As the disaster evolves, the mid phase represents periods of severe impact, where buildings experience substantial deformation or collapse. In the late phase, the scene gradually stabilizes, and the affected areas are mainly characterized by debris accumulation and post-disaster remnants. Multiple video frames were available for each disaster phase. To evaluate the cross-phase consistency of the proposed building extraction method across different disaster stages, we randomly selected three frames per phase to construct the experimental dataset, with two used for training and one for testing. Specifically, frames 160 and 200 were used for training and 165 for testing in the early phase; frames 260 and 295 for training and 290 for testing in the mid phase; and frames 420 and 430 for training and 425 for testing in the late phase. The experimental data are shown in Figure 4.

### 3.2. Evaluation Metrics

To quantitatively evaluate the performance of building extraction from multi-phase disaster imagery, four commonly used evaluation metrics in semantic segmentation were employed: Intersection over union (*IoU*), overall accuracy (*OA*), the *F1-score*, and Boundary F1 (*BF*). *IoU* measures the spatial overlap between the predicted segmentation and the ground truth for a specific class; *OA* reflects the overall pixel-level classification accuracy, and the *F1-score* provides a balanced evaluation by jointly considering precision and recall. All three metrics are computed based on the elements of the confusion matrix. In addition, *BF* evaluates boundary precision by computing the *F1-score* on boundary pixels extracted via morphological operations, where $B_{pred}$ and $B_{gt}$ denote the predicted and ground-truth boundary pixel sets, respectively.

(29) $$IoU=\frac{TP}{TP+FP+FN}$$

(30) $$OA=\frac{TP+TN}{TP+TN+FP+FN}$$

(31) $$F1-score=2\times \frac{Precision\times Recall}{Precision+Recall}$$

(32) $$Precision=\frac{TP}{TP+FP},\;\;\;\;Recall=\frac{TP}{TP+FN}$$

(33) $$BF=\frac{2\times {Precision}_{b}\times {Recall}_{b}}{{Precision}_{b}+{Recall}_{b}}$$

(34) $$Precision_{b}=\frac{\left|B_{pred}\cap B_{gt}\right|}{\left|B_{pred}\right|},\;\;\;\;Recall_{b}=\frac{\left|B_{pred}\cap B_{gt}\right|}{\left|B_{gt}\right|}$$

where TP, FP, TN, and FN represent true positive samples, false positive samples, true negative samples, and false negative samples, respectively.

### 3.3. Implementation Details

All experiments were performed on a single NVIDIA GeForce RTX 3090 Ti GPU using the PyTorch 2.0.1 framework. The AdamW optimizer was adopted with an initial learning rate of 6 × 10^−4^, and the learning rate was scheduled using a cosine annealing strategy.

For the disaster building dataset, images were randomly cropped into 512 × 512 patches. During training, several data augmentation strategies were applied, including random scaling ([0.5, 0.75, 1.0, 1.25, 1.5]), random flipping, random rotation (90°, 180°, 270°), and mosaic augmentation with a ratio of 0.25. The model was trained for 60 epochs with a batch size of 16. During testing, test-time augmentation with horizontal and vertical flipping was employed.

### 3.4. Experimental Results and Analysis

To comprehensively evaluate the effectiveness of the proposed MSS-MambaNet for building extraction in the natural disaster scene, ten representative semantic segmentation networks were selected for comparison, covering three mainstream architectural paradigms in remote sensing image analysis. The first group consists of CNN-based networks, including ABCNet [45], A2FPN [46], and MANet [47], which primarily rely on convolutional feature extraction, enhanced by multi-scale aggregation and attention mechanisms for improved spatial representation. CNN–Transformer hybrids integrate convolutional backbones with Transformer-based global context modeling to capture both fine-grained details and long-range dependencies. Architectures such as UNetFormer [48], BANet [49], and CMTFNet [50] exemplify this design. The third group is recent Mamba-based models, for example, CM-UNet [51], MFMamba [52], UMFormer [53], and GLVMamba [54]. By introducing state-space representations into segmentation networks, these models achieve efficient long-range spatial dependencies with low computational cost.

Under these settings, comprehensive experiments are conducted from multiple perspectives, including qualitative and quantitative comparisons with other methods, which are first presented to assess segmentation accuracy. Subsequently, a cross-phase analysis is carried out to investigate the performance consistency of different models under different disaster stages. Finally, computational complexity comparison and ablation studies are performed to further analyze the efficiency and effectiveness of the proposed architecture.

#### 3.4.1. Overall Performance Comparison

**Figure 5:**
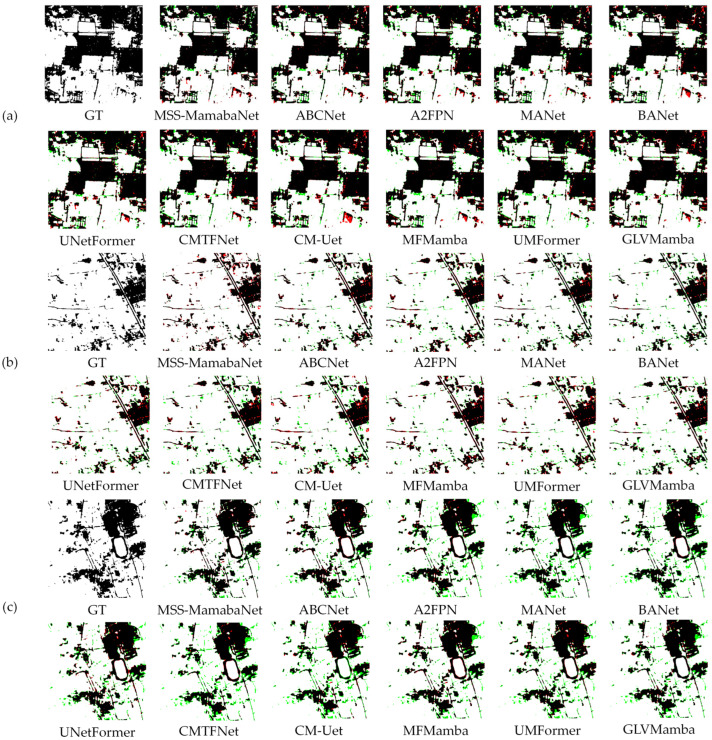
Qualitative comparison of building extraction results across different disaster phases: (**a**) early phase, (**b**) mid phase, and (**c**) late phase. Green pixels indicate false negatives, and red pixels indicate false positives.

To provide an intuitive understanding of the segmentation performance, qualitative comparisons are presented through the visualization of representative samples across all three disaster phases, as illustrated in Figure 5. In the error maps, green pixels indicate false negatives, while red pixels indicate false positives, enabling a direct comparison of missed building regions and background misclassifications among different methods.

In the early phase, buildings remain largely intact, but slender building structures and narrow architectural edges still pose challenges for accurate boundary delineation. As shown in Figure 5a, several baseline methods, especially ABCNet, A2FPN, and MANet, exhibit scattered false negatives along thin building contours and elongated structures, indicating that fine architectural details are easily missed. By contrast, MSS-MambaNet preserves these slender structures more completely and produces cleaner, more contiguous building boundaries with fewer error pixels. During the mid-phase, partial building collapse disrupts structural continuity and makes fragmented building regions harder to identify. As shown in Figure 5b, most competing methods suffer from obvious false negatives on damaged building fragments, while some Transformer-based methods also introduce false positives in debris regions. MSS-MambaNet shows fewer missed detections on discontinuous building parts and maintains lower overall error responses. In the late phase, buildings are severely fragmented and mixed with widespread debris, leading to the most serious omission errors. As observed in Figure 5c, most methods miss a large number of sparse building remnants and generate scattered false positives in chaotic backgrounds. MSS-MambaNet retains a stronger ability to recover dispersed building pixels, with visibly fewer false negatives on small remnants and more compact error distributions, resulting in segmentation results closest to the ground truth under highly degraded scene conditions.

To comprehensively evaluate the effectiveness of the proposed method, quantitative comparisons with ten representative segmentation networks were conducted across early-, mid-, and late-phase disaster scenarios, as summarized in Table 1, Table 2 and Table 3. In the early phase, where buildings remain largely intact but contain many slender structures and fine boundary details, the proposed method attains the highest mIoU of 93.20%, exceeding the second-best method, ABCNet, by 1.02 percentage points. Similar advantages are observed in *OA* and mF1, which reach 96.60% and 96.48%, respectively. For the building category, MSS-MambaNet achieves a Building *IoU* of 91.98%, outperforming the strongest CNN-based baseline, ABCNet, by 1.24 percentage points and the best Mamba-based baseline, MFMamba, by 2.81 percentage points. More importantly, the proposed method achieves the highest Boundary F1 of 95.58%, surpassing ABCNet by 2.36 percentage points and MANet by 2.70 percentage points.

As the disaster progresses into the mid-phase, scene complexity increases substantially due to partial collapse, occlusion, and discontinuous building structures. Despite these challenges, MSS-MambaNet achieves the highest mIoU of 92.27%, exceeding the best competing method, BANet, by 2.19 percentage points. Compared with the strongest Mamba-based baseline, GLVMamba, the proposed method improves mIoU by 3.79 percentage points. For the building category, MSS-MambaNet obtains a Building *IoU* of 91.23%, surpassing ABCNet and BANet by 2.56 and 2.59 percentage points, respectively, as shown in Table 2. Notably, MSS-MambaNet achieves the highest *BF* of 94.09%, outperforming the closest baseline, MANet, by 6.45 percentage points. This large gain suggests that the proposed method is more effective in recovering discontinuous building contours and reducing missed detections in partially collapsed regions, rather than simply producing smoother boundaries.

In the late phase, debris accumulation and highly fragmented building remnants introduce severe semantic ambiguity, making building extraction the most difficult among the three stages. As shown in Table 3, MSS-MambaNet still achieves the best overall performance, with 92.76% mIoU, 96.63% *OA*, and 96.23% mF1. Compared with the strongest baseline in terms of mIoU, ABCNet, the proposed method improves performance by 1.51 percentage points. For the building category, MSS-MambaNet obtains a Building *IoU* of 90.49%, exceeding ABCNet and BANet by 1.93 and 2.22 percentage points, respectively. In addition, MSS-MambaNet achieves the highest *BF* of 92.26%, outperforming BANet by 5.07 percentage points. This improvement indicates that the proposed method can better retain sparse building remnants and suppress debris-induced interference in highly degraded scenes, thereby reducing omission errors on small fragmented targets while maintaining reliable boundary localization.

#### 3.4.2. Performance Across Disaster Phase

The early, mid, and late phases can be regarded as a phase-level progression from relatively easy to difficult extraction conditions. Buildings in the early phase remain relatively complete, partially damaged buildings in the mid phase introduce structural discontinuity and boundary ambiguity, and fragmented remnants in the late phase make building extraction the most challenging. To further evaluate the cross-phase consistency of different methods under progressive structural degradation, the cross-phase performance is analyzed from two complementary perspectives. First, a category-wise comparison is conducted to examine how different architectural paradigms perform across the early, mid, and late disaster phases. Second, a phase-wise dynamic analysis is performed to quantify the performance variation in each method between consecutive phases, thereby assessing cross-phase stability under increasing scene complexity.

**Figure 6:**
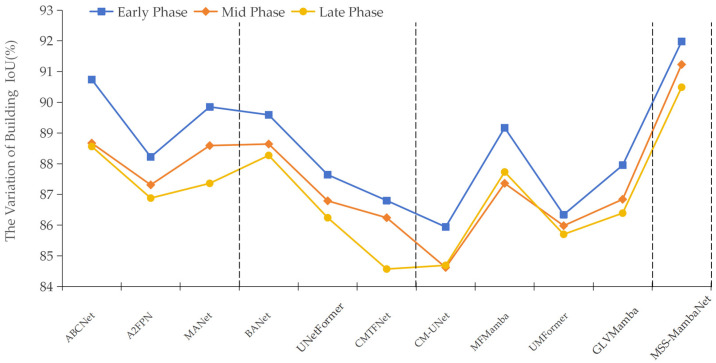
Cross-phase comparison of Building *IoU* for different methods across the early, mid, and late disaster phases.

Figure 6 presents the cross-phase statistical comparison of building extraction performance for all evaluated methods in terms of Building *IoU*. All methods generally achieve the highest accuracy in the early phase, while MSS-MambaNet consistently achieves the highest accuracy across all phases.

From a category perspective, conventional CNN-based encoder–decoder models exhibit relatively strong and stable performances across all disaster phases. In the early disaster phase, when building structures remain visually intact. Among these methods, ABCNet achieves the highest early-phase accuracy with a Building *IoU* of 90.74%, followed by MANet and A2FPN. Their performance remains competitive in the late phases, with most models maintaining Building *IoU* values above 86%. For example, ABCNet still achieves 88.56% in the late phase, while MANet reaches 87.36%. Compared with purely convolutional models, CNN–Transformer hybrid networks show greater variability across architectures. While BANet maintains competitive performance across all phases, other models, such as UNetFormer and CMTFNet, exhibit notably lower accuracy in the late phase. This suggests that although the hybrid design enables effective integration of local and global features, the absolute performance of this category remains dependent on specific architectural implementations. Mamba-based state-space models exhibit substantial variability in absolute performance across architectures. Among them, MFMamba achieves the strongest results within this category, whereas CM-UNet and UMFormer show comparatively lower and more fluctuating accuracy. These observations indicate that although state-space modeling offers advantages in long-range dependency learning, stable structural representation across disaster phases remains challenging. In contrast, MSS-MambaNet consistently achieves the highest Building *IoU* across all phases, with values of 91.98%, 91.23%, and 90.49% from early to late stages, respectively. This superior cross-phase performance demonstrates the effectiveness of the proposed architecture in jointly modeling global context, local structural details, and progressive degradation characteristics.

**Figure 7:**
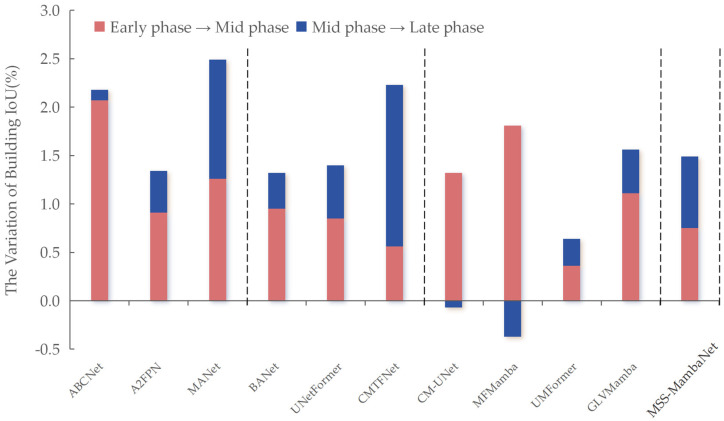
Phase-wise Building *IoU* variation in different methods between consecutive phases. Positive values indicate a performance decrease from an earlier phase to a later phase, while negative values indicate an improvement.

To further investigate cross-phase stability, Figure 7 illustrates the changes in Building *IoU* between consecutive disaster stages. Overall, most methods experience larger performance drops from the early to the mid phase than from the mid to the late phase, indicating that the initial structural disruption of buildings constitutes the dominant source of segmentation difficulty. CNN-based models generally show a monotonic degradation pattern, with performance decreasing consistently across both transitions. However, their degradation is often phase-imbalanced. For example, ABCNet suffers a pronounced *IoU* reduction in the early-to-mid transition, followed by a relatively smaller drop in the subsequent stage, showing the limited adaptability to progressively fragmented scenes. CNN–Transformer hybrid networks generally exhibit smoother phase-to-phase transitions than purely CNN-based models, although notable intra-category differences remain. While BANet and UNetFormer maintain balanced performance drops across both transitions, CMTFNet shows clear late-phase sensitivity, indicating increased vulnerability to severe fragmentation and semantic ambiguity. In contrast, Mamba-based models exhibit the most diverse and less predictable transition behaviors among all categories. Although their overall trend remains downward, adjacent-stage variations differ substantially across architectures, with certain methods even showing localized fluctuations. Both CM-UNet and MFMamba display non-monotonic phase-to-phase variations, with MFMamba showing a more evident mid-to-late increase in Building *IoU* than CM-UNet. These results suggest that global sequence modeling alone may be insufficient to ensure stable structural representation throughout disaster progression. Compared with all competing methods, MSS-MambaNet exhibits the most controlled and uniform degradation pattern across disaster phases. Its Building *IoU* decreases by only 0.75 percentage points from the early phase to the mid phase and 0.74 points from the mid phase to the late phase, indicating strong cross-phase consistency. This near-uniform transition behavior indicates the strong cross-phase consistency of the proposed method under progressively degraded disaster conditions.

#### 3.4.3. Comparison of Complexity

To evaluate the computational efficiency of MSS-MambaNet, we analyze model complexity in terms of the number of parameters (Params, in millions), floating-point operations (FLOPs, in billions), inference speed (FPS), and building extraction accuracy (Building mIoU). All models are evaluated under the same experimental settings to ensure a fair comparison. As shown in Table 4, MSS-MambaNet contains 12.37 M parameters, which is comparable to most CNN-, CNN–Transformer, and Mamba-based models. With this moderate parameter scale, it achieves the highest Building mIoU of 92.78% among all compared methods. In terms of FLOPs, MSS-MambaNet requires 42.09 G, which is higher than that of lightweight CNN-based models such as ABCNet and A2FPN. However, this additional computational cost is accompanied by clear accuracy gains, with mIoU improvements of 1.60 and 3.11 percentage points over ABCNet and A2FPN, respectively. Compared with larger or computationally heavier methods, such as MANet, BANet, UNetFormer, and UMFormer, MSS-MambaNet achieves higher accuracy with fewer parameters or comparable computational complexity. In addition, compared with recent Mamba-based models, MSS-MambaNet improves mIoU by 2.65–5.13 percentage points while maintaining a moderate model size. Overall, MSS-MambaNet achieves the highest segmentation accuracy with a moderate model size and acceptable computational cost, indicating a favorable trade-off between accuracy and efficiency.

#### 3.4.4. Ablation Study

To quantitatively validate the effectiveness of each proposed component in our framework, a comprehensive ablation study was conducted. We incrementally integrated the Pixel-Aware Dynamic Weighting Loss Function, the DDCGF module, and the MSS-Mamba backbone to isolate their individual and synergistic contributions. As summarized in Table 5, the models’ performances, calculated as the average across all three disaster phases, demonstrate a clear and progressive improvement in building extraction performance, highlighting a favorable balance between accuracy and efficiency. These ablation results indicate that the proposed modules are complementary rather than redundant. PADW improves optimization under phase-dependent imbalance and boundary ambiguity, DDCGF enhances spatial-frequency feature interaction, and MSS-Mamba strengthens multi-scale global–local representation. Their combined use leads to the best overall performance, confirming that the proposed framework provides more than an incremental combination of existing components.

(1) Effectiveness of the Pixel-Aware Dynamic Weighting Loss Function

The Pixel-Aware Dynamic Weighting Loss serves as the first enhancement over the baseline by addressing the severe class imbalance and boundary ambiguity in disaster scenes. As shown in Table 5, introducing this loss alone improves the mIoU from 91.33% to 91.96% and the *OA* from 95.68% to 96.00%, with the Building *IoU* increasing by 0.77 percentage points. These gains indicate that reweighting the optimization process toward underrepresented building pixels helps the model learn more discriminative representations for minority classes.

To further isolate the contribution of PADW, we compare it against five widely used loss functions under the identical MSS-MambaNet architecture. As shown in Table 6, PADW achieves the highest performance across all four metrics: *OA* of 96.43 percent, mF1 of 96.25 percent, *BF*-Score of 93.98 percent, and mIoU of 92.78 percent. The most pronounced gain is in boundary quality, where PADW surpasses the next best alternative (Edge loss, *BF*-Score 91.78 percent) by 2.20 percentage points. Compared with the standard Cross-Entropy loss, PADW improves mIoU by 1.20 points and *BF*-Score by 2.80 points. These results confirm that the phase-aware dual weighting mechanism provides benefits beyond what standard loss functions can offer, particularly in handling boundary-ambiguous pixels in degraded disaster scenes.

(2) Effectiveness of DDCGF Module

Building on the improved optimization strategy, the DDCGF module further enhances feature representation by enabling cross-domain interaction between spatial and frequency information. As reported in Table 5, adding DDCGF improves mIoU by an additional 0.37 percentage points and *OA* by 0.19 points, while the Building *F1-score* increases by 0.26 percentage points. Notably, this improvement is achieved with a slight reduction in model parameters, from 13.01 M to 12.90 M, suggesting that DDCGF improves feature utilization efficiency rather than increasing model complexity.

(3) Effectiveness of MSS-Mamba Backbone

The MSS-Mamba backbone further strengthens the model by introducing multi-scale selective state-space modeling for efficient long-range dependency capture. Incorporating MSS-Mamba raises mIoU from 91.96% to 92.37% and improves *OA* to 96.23%, while reducing the parameter count to 12.48 M. These results confirm that MSS-Mamba enhances global context modeling and structural coherence with a more compact architecture, which is particularly beneficial for natural disaster imagery containing fragmented and heterogeneous building structures.

(4) Synergistic Effect of the Unified Feature Optimization Strategy

When all components are combined, MSS-MambaNet achieves the best overall performance, with an mIoU of 92.78%, an *OA* of 96.43%, and an mF1 of 96.25%, while maintaining only 12.37 M parameters. This result demonstrates that the three components are complementary: the loss function stabilizes optimization under class imbalance, DDCGF improves cross-domain feature refinement, and MSS-Mamba provides efficient multi-scale global modeling. Together, they form a compact and effective framework for consistent building extraction in complex disaster scenes.

## 4. Discussion

This work presents MSS-MambaNet, a Multi-Scale Mamba-based architecture for building extraction from multi-phase disaster imagery. Unlike video-based temporal modeling methods, MSS-MambaNet does not explicitly model inter-frame temporal consistency or damage evolution trajectories across consecutive frames. Instead, it focuses on cross-phase building extraction from discrete early-, mid-, and late-stage disaster imagery, where building appearances vary from intact structures to partially damaged buildings and fragmented remnants. To address these cross-phase distribution shifts, the proposed method integrates efficient long-range dependency modeling, cross-domain feature discrimination, and phase-aware adaptive optimization within a unified learning framework. Extensive experiments across early, mid, and late disaster phases demonstrate that MSS-MambaNet consistently outperforms state-of-the-art CNN-, CNN–Transformer, and Mamba-based methods, achieving superior segmentation accuracy with a moderate model size. The results further indicate its consistent performance under severe structural variation, occlusion, and dynamic scene complexity.

It is important to note that, in this work, the definition of “building” is intentionally extended to include not only intact structures but also partially damaged and highly fragmented remnants. This unified representation enables consistent tracking of structural evolution throughout the disaster lifecycle, supporting stable building extraction under progressive degradation. The current framework does not explicitly distinguish different damage levels, as it primarily focuses on robust structural extraction rather than fine-grained damage assessment.

Future work will extend this framework toward damage-level classification and more refined semantic categorization, enabling a more comprehensive understanding of building conditions in dynamic disaster scenarios. It should also be acknowledged that the current validation is based on a single satellite video of the Petobo tsunami event. Therefore, the present experiments mainly demonstrate the model’s consistent performance across early, mid, and late phases within this specific disaster event, rather than fully proving generalizability across different disasters, sensors, cities, or imaging conditions. Further validation on additional disaster events, satellite sensors, and geographic regions will be pursued in future work.

## Figures and Tables

**Table 1:** Quantitative comparison of segmentation performance in the early phase.

Type	Method	*F1* (%)		*IoU* (%)		Evaluation Index			
		Other	Building	Other	Building	*OA* (%)	mF1 (%)	*BF* (%)	mIoU (%)
**CNN** **-based**	**ABCNet (2021)**	96.70	95.15	93.61	90.74	96.07	95.92	93.22	92.18
	**A2FPN (2021)**	95.76	93.74	91.87	88.22	94.95	94.75	90.34	90.05
	**MANet (2022)**	96.39	94.66	93.02	89.85	95.69	95.52	92.88	91.44
**CNN-** **Transformer**	**BANet (2021)**	96.26	94.51	92.79	89.59	95.55	95.38	91.95	91.19
	**UNetFormer (2022)**	95.52	93.41	91.42	87.64	94.67	94.47	91.42	89.53
	**CMTFNet (2023)**	95.26	92.93	90.95	86.80	94.33	94.10	88.07	88.87
**Mamba** **-based**	**CM-UNet (2024)**	94.78	92.44	90.07	85.94	93.82	93.61	87.46	88.00
	**MFMamba (2025)**	96.08	94.28	92.46	89.17	95.35	95.18	89.13	90.82
	**UMFormer (2025)**	95.10	92.67	90.67	86.34	94.13	93.89	88.63	88.50
	**GLVMamba (2025)**	95.58	93.59	91.54	87.95	94.77	94.59	89.07	89.75
**Ours**	**MSS-MambaNet**	97.13	95.82	94.43	91.98	96.60	96.48	95.58	93.20

**Table 2:** Quantitative comparison of segmentation performance in the mid-phase.

Type	Method	*F1* (%)		*IoU* (%)		Evaluation Index			
		Other	Building	Other	Building	*OA* (%)	mF1 (%)	*BF* (%)	mIoU (%)
**CNN** **-based**	**ABCNet (2021)**	95.46	93.99	91.31	88.67	94.83	94.73	86.41	89.99
	**A2FPN (2021)**	94.93	93.22	90.35	87.31	94.20	94.08	85.09	88.83
	**MANet (2022)**	95.53	93.95	91.45	88.59	94.86	94.74	87.64	90.02
**CNN-** **Transformer**	**BANet (2021)**	95.58	93.98	91.53	88.64	94.90	94.78	87.37	90.08
	**UNetFormer (2022)**	94.70	92.93	89.93	86.79	93.94	93.81	86.94	88.36
	**CMTFNet (2023)**	94.59	92.61	89.73	86.24	93.75	93.60	82.60	87.98
**Mamba** **-based**	**CM-UNet (2024)**	93.86	91.67	88.43	84.62	92.93	92.77	81.44	86.53
	**MFMamba (2025)**	94.90	93.25	90.29	87.36	94.19	94.08	83.36	88.83
	**UMFormer (2025)**	94.34	92.46	89.29	85.98	93.54	93.40	83.55	87.64
	**GLVMamba (2025)**	94.80	92.96	90.12	86.84	94.02	93.88	84.56	88.48
**Ours**	**MSS-MambaNet**	96.53	95.42	93.30	91.23	96.05	95.98	94.09	92.27

**Table 3:** Quantitative comparison of segmentation performance in the late phase.

Type	Method	*F1* (%)		*IoU* (%)		Evaluation Index			
		Other	Building	Other	Building	*OA* (%)	mF1 (%)	*BF* (%)	mIoU (%)
**CNN** **-based**	**ABCNet (2021)**	96.88	93.93	93.94	88.56	95.87	95.40	85.49	91.25
	**A2FPN (2021)**	96.44	92.98	93.12	86.88	95.27	94.71	83.88	90.00
	**MANet (2022)**	96.60	93.25	93.42	87.36	95.48	94.92	86.48	90.39
**CNN-** **Transformer**	**BANet (2021)**	96.83	93.77	93.85	88.27	95.80	95.30	87.19	91.06
	**UNetFormer (2022)**	96.27	92.61	92.81	86.24	95.04	94.44	84.75	89.52
	**CMTFNet (2023)**	95.85	91.64	92.02	84.57	94.45	93.74	79.46	88.29
**Mamba** **-based**	**CM-UNet (2024)**	95.78	91.71	91.90	84.69	94.41	93.74	81.74	88.29
	**MFMamba (2025)**	96.66	93.47	93.54	87.73	95.58	95.06	84.47	90.64
	**UMFormer (2025)**	96.13	92.30	92.55	85.70	94.85	94.22	81.75	89.13
	**GLVMamba (2025)**	96.34	92.70	92.94	86.39	95.12	94.52	83.93	89.66
**Ours**	**MSS-MambaNet**	97.45	95.01	95.03	90.49	96.63	96.23	92.26	92.76

**Table 4:** Comparison of computational complexity and segmentation performance among different methods.

Type	Method	Params (M)	FLOPs (G)	FPS	mIoU (%)
**CNN** **-based**	**ABCNet (2021)**	13.52	7.81	118.3	91.18
	**A2FPN (2021)**	12.66	9.60	96.7	89.67
	**MANet (2022)**	57.49	102.96	15.2	90.67
**CNN-** **Transformer**	**BANet (2021)**	30.07	33.00	41.8	90.82
	**UNetFormer (2022)**	35.86	35.68	29.4	89.19
	**CMTFNet (2023)**	11.72	50.84	21.6	88.45
**Mamba** **-based**	**CM-UNet (2024)**	13.88	12.01	96.0	87.65
	**MFMamba (2025)**	11.27	19.52	58.3	90.13
	**UMFormer (2025)**	12.38	47.70	25.1	88.47
	**GLVMamba (2025)**	24.19	7.67	132.6	89.35
**Ours**	**MSS-MambaNet**	12.37	42.09	39.8	92.78

**Table 5:** The results of the ablation experiments of the MSS-MambaNet. “**✓**” indicates used, and “**✗**” indicates not used.

PADW Loss	DDCGF	MSS- Mamba	Param (M)	*F1* (%)		*IoU* (%)		Evaluation Index		
				Other	Building	Other	Building	*OA* (%)	mF1 (%)	mIoU (%)
**✗**	**✗**	**✗**	13.01	96.46	94.46	93.16	89.51	95.68	95.46	91.33
**✓**	**✗**	**✗**	13.01	96.72	94.89	93.64	90.28	96.00	95.81	91.96
**✓**	**✓**	**✗**	12.90	96.86	95.15	93.91	90.75	96.19	96.01	92.33
**✓**	**✗**	**✓**	12.48	96.92	95.13	94.02	90.72	96.23	96.03	92.37
**✓**	**✓**	**✓**	12.37	97.06	95.44	94.29	91.28	96.43	96.25	92.78

**Table 6:** Performance comparison of different loss functions under the same MSS-MambaNet architecture.

Loss Function	*OA* (%)	mF1 (%)	*BF* (%)	mIoU (%)
**Cross-Entropy**	95.82	95.6	91.18	91.58
**Dice**	95.68	95.46	90.8	91.33
**Focal**	95.65	95.42	90.86	91.26
**Focal + Dice**	95.83	95.63	91.59	91.64
**Edge**	95.8	95.6	91.78	91.58
**PADW**	96.43	96.25	93.98	92.78

## Data Availability

The original contributions presented in this study are included in the article; further inquiries can be directed to the corresponding author.
